# Prophylactic mesh reinforcement in elective abdominal surgeries: a systematic review, meta-analysis, and GRADE evidence assessment

**DOI:** 10.1007/s10029-025-03421-9

**Published:** 2025-07-11

**Authors:** Ahmed W. Abbas, Mohamed F. Abo-elsoad, Mahmoud Diaa Hindawi, Mohamed Abo Zeid, Abd-Elfattah Kalmoush, Menna M. Aboelkier, Mohamed A. Aldemerdash, Rashad G. Mohamed, Hosam Elghadban

**Affiliations:** 1 Faculty of Medicine, Mansoura University, Mansoura, Egypt; 2 Faculty of Medicine, Al-Azhar University, Cairo, Egypt; 3 Faculty of Medicine, Tanta University, Tanta, Egypt; 4 Master program, Faculty of Science, Cairo University, Cairo, Egypt; 5 Faculty of Medicine, Sohag University, Sohag Al Gadida City, Egypt; 6 Mansoura Manchester Program for Medical Education, Faculty of Medicine, Mansoura University, Mansoura, Egypt; 7 General Surgery Department, Faculty of Medicine, Mansoura University, Mansoura, Dakahlia Egypt

**Keywords:** MESH, Elective laparotomy, Abdominal wall closure, Incisional hernia

## Abstract

**Background:**

Elective laparotomies account for a larger fraction of laparotomy procedures performed worldwide. Although surgical techniques continue to advance, the incidence of incisional hernia (IH) and other post-operative complications remain challenging to surgeons. This study aimed to evaluate the significance of using prophylactic mesh reinforcement during elective laparotomy.

**Methods:**

A comprehensive search was conducted in PubMed, Scopus, and Web of Science to identify studies that included adults undergoing elective abdominal surgery and compared prophylactic mesh reinforcement of the abdominal wall using any type of mesh in any anatomical position to standard fascial closure with sutures alone, without mesh. The analysis aimed to assess the impact of mesh reinforcement on the incidence of IH at all possible timepoints, in addition to secondary outcomes based on mesh technique, such as wound infections, dehiscence, seroma, re-operation for IH, and prolonged hospital stay. Data analysis was performed using the R programming language.

**Results:**

Fifteen RCTs, including 2,233 patients with follow-up durations ranging from 1.5 to 5 years, were analyzed. Prophylactic mesh reinforcement significantly reduced the incidence of IH following elective gastrointestinal surgeries at 12 months (risk ratio [RR] = 0.35, 95% confidence interval [CI] [0.14; 0.86], *p* = 0.02), 24 months (RR = 0.28, 95% CI [0.11; 0.68], *p* < 0.01), 36 months (RR = 0.62, 95% CI [0.36; 1.06], *p* = 0.08), and 48 months (RR = 0.35, 95% CI [0.11; 1.17], *p* = 0.09). Similarly, mesh significantly reduced IH rates following open abdominal aortic aneurysm repair at 12 months (RR = 0.13, 95% CI [0.04; 0.41], *p* < 0.01), 24 months (RR = 0.31, 95% CI [0.21; 0.45], *p* < 0.01), and 36 months (RR = 0.23, 95% CI [0.10; 0.54], *p* < 0.01).

**Conclusions:**

Prophylactic mesh reinforcement during elective abdominal laparotomy significantly reduced the incidence of IH and the need for reoperation. However, it is associated with an increased risk of seroma formation and, to a lesser extent, wound infection, particularly with the Sublay technique.

**Supplementary Information:**

The online version contains supplementary material available at 10.1007/s10029-025-03421-9.

## Introduction

Incisional hernia (IH) occurs in about 10 to 15 percent of patients who have had a previous abdominal incision [1]. This type of hernia can develop following any surgical incision, including midline, paramedian, subcostal, McBurney, Pfannenstiel, and flank incisions [2]. The highest rate of occurrence following the midline incisions ranges from 3 to 20 percent [3]. It has previously been suggested that midline incisions should be reserved for emergency surgery and other surgical procedures for which the entire abdominal cavity should be accessible [4].

In a multicenter randomized trial, the incidence of IH was significantly lower in the group with continuously used nylon compared to continuous polyglactin 910 (10.3% vs. 20.6%), although no statistically significant differences were found for wound dehiscence [5].

The prevention of abdominal wound dehiscence and/or IH by using preventive abdominal binders is highly surgeon-dependent. The effects of these medical aids have been disputed and should be considered carefully and weighed against the potential risks of lung atelectasis and possible pneumonia [6].

Placement of mesh to prevent IH has primarily been investigated in high-risk patient groups, such as patients with abdominal aortic aneurysms (AAA) and obesity, in whom the incidences of IH of up to 38% and 50%, respectively [7].

Synthetic mesh is associated with low hernia recurrence rates but may increase the risk of surgical site and mesh infections. It is permanent and can lead to long-term complications [8]. Biologic mesh has traditionally been used in contaminated or emergency cases [9]. A randomized trial found no difference in abdominal wall complications between mesh placement compared to no mesh, but complications necessitating reoperation were more common with biologic mesh [10]. Moreover, we have to keep in mind that the technique of fascial closure significantly impacts IH prevention and hospital stay, in both elective and emergency laparotomy settings, as highlighted in recent findings [11–13].

Data on prophylactic bioabsorbable mesh indicates that it is effective at preventing hernias during the first postoperative year, with no increase in surgical complications; however, long-term results are not yet available [14]. Prophylactic mesh reinforcement can be applied using different anatomical techniques, most commonly the onlay and sublay approaches. In the onlay technique, the mesh is placed over the anterior fascia of the abdominal wall, above the rectus sheath. In contrast, the sublay technique involves positioning the mesh in a deeper plane, between the rectus muscle and the posterior rectus sheath or peritoneum. Each approach aims to support fascial closure and reduce the risk of incisional hernia, though they differ in complexity and potential complication profiles [15].

Prophylactic mesh has not reached the point of widespread use, especially in elective laparotomies. This study was conducted to review the published studies on elective abdominal surgery utilizing mesh reinforcement as a prophylactic approach to assess safety, feasibility, the incidence of IH, and whether this incidence is affected by the cause of elective laparotomy (GIT (gastrointestinal) cause/open AAA cause), and postoperative complications.

## Methods

Our approach and results conformed strictly to the guidelines of meta-analysis and systematic review, including the utilization of PRISMA 2020 [16] and the Cochrane handbook [17]. To maintain transparency, we recorded our protocol on the Open Science Framework (OSF) with the corresponding DOI (https://doi.org/10.17605/OSF.IO/P3XQW).

### Literature search

We conducted a comprehensive literature search using PubMed, SCOPUS, and Web of Science covering publications up to May 2025. The aim was to identify studies demonstrating the efficacy of prophylactic mesh in reducing the incidence of IH, as well as any associated adverse events following elective laparotomy. Our search strategy included relevant terms such as “Mesh,” “Abdominal closure,” “Laparotomy,” and “Elective” to ensure a thorough and targeted review of the available evidence. The detailed search strategy for each database is demonstrated in ESM. 1.

### Eligibility criteria and study selection

We included randomized controlled trials (RCTs) that evaluated the use of prophylactic mesh reinforcement during elective surgery in adult patients. Eligible studies compared mesh augmentation against non-mesh-based abdominal fascial closure, broadly classified here as"conventional closure techniques."These typically involved continuous or interrupted suturing using absorbable or non-absorbable monofilament sutures (e.g., polypropylene, polyglactin, or polydioxanone), without the use of mesh or additional reinforcement. Rather than categorizing trials by specific suture technique or specific mesh type used, our analysis focused on the more clinically relevant comparison of mesh versus non-mesh, reflecting the continuous evolution of evidence in the field; however, we extracted and summarized all these aspects from the included studies. This approach aligns with the trajectory of prior meta-analyses, as summarized in Table 4, and advances the literature by integrating granular subgroup assessments by clinical context and mesh placement.

To guide our study selection, we applied the PICOS framework:

- **Population (P):** Adults undergoing elective abdominal surgery.
- **Intervention (I):** Prophylactic mesh reinforcement of the abdominal wall, using any type of mesh or, in any position
- **Comparator (C):** Standard fascial closure with sutures alone, without mesh.
- **Outcomes (O):** Incidence of incisional hernia (IH) at 12, 24, 36, and 48 months; and postoperative complications according to the mesh technique (only or sublay), including wound infection, seroma, hematoma, wound dehiscence, reoperation for IH, abdominal pain, and duration of hospitalization.
- **Study Design (S):** Randomized controlled trials published in English.

### Exclusion criteria

During the title and abstract screening phase, we excluded all studies that met any of the following criteria: non-randomized trials, observational studies, review articles, animal studies, non-English publications, letters to the editor, conference abstracts, editorials, commentaries, and unpublished works, including dissertations, theses, and other forms of gray literature.

At the full-text screening stage, additional exclusion criteria were applied to ensure alignment with our study objectives and outcome measures. Specifically:

- Trials involving emergency laparotomies were excluded. In studies that included both elective and emergency cases, only data from the elective subgroup were extracted and analyzed.
- Studies including pregnant participants were excluded due to the established association between pregnancy and an increased risk of incisional hernia, which could act as a confounding variable when evaluating mesh efficacy.
- Initially, we planned to exclude studies enrolling patients with a BMI ≥ 27 kg/m2, referencing evidence from the INSECT trial [18], which showed a 20% risk of incisional hernia in this population within one year postoperatively. However, this exclusion criterion was ultimately dropped due to inconsistent BMI reporting across trials and the wide variability in BMI thresholds used.

Any disagreements in study selection were resolved through consensus or by consultation with a senior leader.

### Quality assessment

The risk of bias (ROB) of all included studies was precisely assessed through 2 blinded reviewers (RG and MAE) using the ROB 2 tool [19]. This tool is composed of five main domains: randomization process, deviation from the intended interventions, missing outcome data, measurement of the outcome, and selection of the reported result. Authors'judgments for each domain were grouped as ‘Low risk,’ ‘Some concerns,’ or ‘High risk’ of bias. A third expert author discussed and resolved any differences in data extraction and bias assessment.

### Data extraction and study outcomes

Data extraction was conducted by two independent reviewers (MFA and MA) using a standardized form. Extracted data included in the summary of studies’ data table (Table 1) houses the study design, sample size, population characteristics, intervention and comparator details, cause of laparotomy, follow-up duration, and position of MESH. Besides, the studies’ baseline and outcome data extracted were as follows: Age, gender, smoking status, surgery duration, history of previous laparotomy, previous IH, blood transfusion, and comorbidities, mainly diabetes, cardiovascular, and chronic obstructive pulmonary disease (COPD). Furthermore, an additional 2 tables were performed for the investigation of different causes of GIT laparotomies (Table 3) and the other one for the comparison of the similarities and the differences exhibited in our study and previously published similar meta-analyses (Table 4). Causes of GI laparotomies categorized by anatomical site: These include upper GI (esophagus to the first part of the duodenum), lower GI (second half of the duodenum to the anus), hepatobiliary, pancreatic, and other procedures (such as bariatric surgeries). Additionally, the GI laparotomies were classified based on etiology, distinguishing between neoplastic (tumor-related) and non-neoplastic causes.

**Table 1:** Summary of included studies. *OAAA* open abdominal aortic aneurysm, *GIT* gastrointestinal tract

Study ID	Country/Location	Study design	Follow-up time (years)	Casue of laparotomy	Mesh type	Mesh method	Mesh location	Comparator	Inclusion criteria	Primary Outcome	Conclusion
Abo-ryia 2013	Egypt/Tanta	RCT	4	GIT causes (bariartic surgery)	polypropylene mesh	Sublay	preperitoneal	conventional suture wound closure	All patients were candidates for bariatric surgery in accordance with National Institutes of Health consensus criteria for the management of morbid obesity	operative time, Postoperative wound complications (Infection, incisional hernia, Partial dehiscence and seroma)	Using preperitoneal Prolene mesh for closing wounds in open bariatric surgery is tolerable and efficient in preventing incisional hernia
Bali 2014	Greece	RCT	3	OAAA	bovine pericardium mesh	onlay	NA	routine abdominal suture closure	Patients undergoing elective open AAA without a history of a previous abdominal surgery or receiving medications like steroids or other immunosuppressive drugs	development of incisional hernia after 3 years of surgery	The use of bovine pericardium mesh in patients having elective open AAA repair for closure of fascia demonstrated better outcomes and less complications rate of incisional herniation
Bevis 2010	UK	RCT	3	OAAA	polypropylene mesh	Sublay	preperitoneal	routine abdominal closure	Patients undergoing elective open AAA with or without a history of a previous abdominal surgery	development of incisional hernia after 3 years of surgery	The use of mesh significantly improved the rate of postoperative incisional hernia following open AAA repair without increasing the risk of developing any complications
Brosi 2017 & Glauser 2019	Switzerland	RCT	5	GIT causes	polyester (polyethylene terephthalate) mesh	onlay	Intraperitoneal	routine abdominal closure without mesh	Patients scheduled or median laparotomy with or without a history of a previous laparotomy	Incidence of incisional hernia 2 years after surgery	The use of a non-absorbable prophylactic intraperitoneal onlay mesh can reduce the risk of incisional hernia
Caro 2014 & Caro 2018	Spain	RCT	3	GIT causes	polypropylene mesh	onlay	Supra-Aponeurotic	a standard abdominal wall closure technique	Patients with American Society of Anesthesiologists (ASA) score < 4 who needed a midline laparotomy in elective surgery	Incisional hernia	Applying prophylactic supra-aponeurotic mesh reduces the incidence of incisional hernia regardless of other factors
El-khadrawy 2009	Egypt/Tanta	RCT	3	GIT causes	polypropylene mesh	Sublay	preperitoneal	routine abdominal closure without mesh	High-risk patients liable to develop postoperative incisional hernia	Incisional hernia	Prophylactic subfascial mesh in midline closure in high-risk patients can be both safe and effective in providing strength to the wound to prevent incisional hernia
Garcia-urena 2015	Spain	RCT	2	GIT causes	Polypropylene Mesh	onlay	Over the fascia	routine abdominal closure without mesh	Patients older than 18 years, operated on any colorectal disease (both elective and emergency surgical procedures) through a midline laparotomy	Incidence of incisional hernia during a 2-year postoperative follow-up	The incidence of incisional hernia is high in patients undergoing colorectal surgeries whether elective or emergency. The use of a prophylactic polypropylene mesh on the onlay position improves the rate of incisional hernia without any morbidity
Gutierrez 2003	Spain	RCT	3	GIT causes	polypropylene mesh	onlay	Supra-Aponeurotic	routine abdominal closure without mesh	Patients undergoing a vertical laparotomy with a length exceeding 10 cm, considered to be at high risk for incisional hernia, exhibited at least one of the following characteristics: Surgery due to neoplastic pathology Age over 70 Respiratory failure Clear malnutrition Severe obesity (Body Mass Index over 30) Habitual smoking (more than 20 cigarettes daily)	Incidence of incisional hernia 3 years after surgery	Closing laparotomies with a high risk of incisional hernia using polypropylene mesh is useful for decreasing of the risk of incisional hernias
Honig 2021	Germany	RCT	2	OAAA	polypropylene mesh	onlay	On the rectus fascia	Normal wound closure using either long-term absorbable or extra long-term absorbable synthetic monofilament suture	adults aged 18 years and older with an indication for elective treatment of AAA by median laparotomy	incidence of incisional hernia within 24 months of follow-up	The incidence of incisional hernia showed no significant difference between mesh and primary suture, challenging current guidelines for prophylactic mesh in open AAA repair
Jairam 2017	Austria, Germany, and the Netherlands	RCT	2	OAAA	polypropylene mesh	onlay	On the rectus fascia	Sublay mesh reinforcement and routine abdominal closure without mesh	adults aged 18 years or older who underwent elective midline laparotomy and had either an abdominal aortic aneurysm or a BMI equal to or higher than 27 kg/m^2^	Incisional hernia during 2 years of follow-up	A substantial improvement in rate of incisional hernia was found with onlay mesh reinforcement compared with sublay mesh reinforcement and primary suture only. Onlay mesh can become the standard treatment for high-risk patients undergoing midline laparotomy
Kohler 2019	Switzerland	RCT	3	GIT causes (bariartic surgery)	polypropylene mesh	onlay	Intraperitoneal	routine abdominal closure without mesh	Patients older than 18 undergoing elective surgery with at least two of the following risk factors—BMI over 25, neoplastic disease, male sex, or history of laparotomy—are at higher risk for developing an incisional hernia	Incidence of an incisional hernia	The use of prophylactic intraperitoneal mesh implantation in patients at high risk for incisional hernia was found to reduce the incidence of hernia but with increased risk of pain early postoperatively and prolonged wound healing of surgical site infection
Muysoms 2016	Belgium	RCT	2	OAAA	polypropylene mesh	Sublay	Behind the rectus muscles and anterior to the posterior rectus fascia	routine abdominal closure without mesh	Adult patients planned for elective AAA treatment by a midline laparotomy were eligible	the incidence of incisional hernia at 2-year follow-up	The use of prophylactic mesh-augmented strengthening of a laparotomy in retro muscular region in patients with abdominal aortic aneurysm is safe and effective in preventing the improving the rate of incisional hernia during 2 years, with an extra mean operative time of 16 min
Pans 1998	Belgium	RCT	2.5	GIT causes (bariartic surgery)	polyglactin	onlay	Intraperitoneal	routine abdominal closure without mesh	Patients with morbid obesity	Incidence of incisional hernia	There is no use of using an intraperitoneal polyglactin mesh to prevent incisional hernias in obese patients
Sarr 2014	USA	RCT	2	GIT causes	Surgisis Gold graft	Sublay	under the posterior rectus sheath	routine abdominal closure without Surgisis Gold graft	The study included patients older than 18 years with morbid obesity (BMI > 40 kg/m^2^ or BMI > 35 with weight-related comorbidities) undergoing open bariatric surgery, specifically open RYGB. Patients undergoing reoperative bariatric surgery were included if they were revising a failed previous bariatric procedure and did not have a concomitant incisional hernia. Additionally, patients with a small (< 2.5 cm) nonincarcerated umbilical hernia were included, provided the hernia had not been previously repaired	Rate of incisional hernia 6 weeks, 3, 6, and 9 months, and 1 and 2 years after RYGB	Using Surgisis Gold for strengthening the abdominal wall after open RYGB did not show to be greatly different from a primary suture repair
Strzelczyk 2006	Poland	RCT	2.3	GIT causes (bariartic surgery)	polypropylene mesh	Sublay	between the rectus muscle and its posterior sheath	routine abdominal closure without mesh	Morbid obesity and failure to reduce bodyweight with conventional treatment (diet, exercise, anorectic agents)	Incisional hernia every 6 month	The use of a mesh reduces the risk of hernia development and did not lengthen hospital stay

### Outcome definition

The primary outcome was the incidence of IH defined as any abdominal wall gap with or without a bulge in the area of the postoperative scar perceptible or palpable by clinical examination or imaging (ultrasound (US) and/or computed tomography (CT)), as determined by the European Hernia Society [20]. This outcome data were extracted along the following follow-up durations, beginning from 12 months until 48 months. The secondary outcomes were related to post-operative adverse events, which were as follows: incidence of infection, seroma, hematoma, duration of hospitalization, abdominal pain, and re-operation for IH. The secondary outcomes were clinically assessed by the masked physician, except for the abdominal pain, which was self-assessed by using a pain score pre-specified before the start of the trial.

### Data synthesis and heterogeneity assessment

We used R statistical software (version 2024.04.2) and the ‘meta’ package (version 7.0–0) [21] for the analysis. We computed risk ratios (RR) for dichotomous outcomes and mean differences (MD) for continuous outcomes employing a random-effect model. DerSimonian Laird's random effects model produced pooled estimates with a greater standard error to accommodate for any inconsistent pooled effect sizes, which were used for all outcomes. Since those estimates were cautious, any potential inconsistencies in our computed meta-analysis effect sizes must be taken into account. Visual inspection of the forest plots was used to assess statistical heterogeneity between trials, and the Chi-square test (also known as the Cochrane Q test) and Higgins and Thompson I2, which has the formula I2 = ((Q-df)/Q) × 100%, were used to quantify it. If the chi-square test's p-value was less than 0.1, the statistical heterogeneity between studies was considered to be significant, and heterogeneity was considered to be low, moderate, and high if I2 was < 25%, from 25–75%, or > 75%, respectively. In cases where heterogeneity was observed, a random-effect model was utilized. Subgroup analysis was completed considering various factors, including the cause of elective abdominal laparotomy in the incidence of IH and the site of mesh in hematoma, seroma, and wound infection. The significance was determined by the 95% confidence interval (CI) [22]. Sensitivity analyses were carried out using the leave-one-out model.

### GRADE assessment

We assessed the quality of evidence using the Grading of Recommendations Assessment, Development, and Evaluation (GRADE) framework. We evaluated the quality of the primary outcomes: incidence of IH all time points (12, 24, 36, and 48 months) after open AAA and GIT surgeries. Disagreements were resolved through consultation with a third author [23].

### Publication bias assessment

We attempted to assess publication bias using a funnel plot, but this was not statistically feasible due to the insufficient number of studies, as at least ten studies are required according to Egger et al. [24].

## Results

### Characteristics of the included studies

**Fig. 1:**
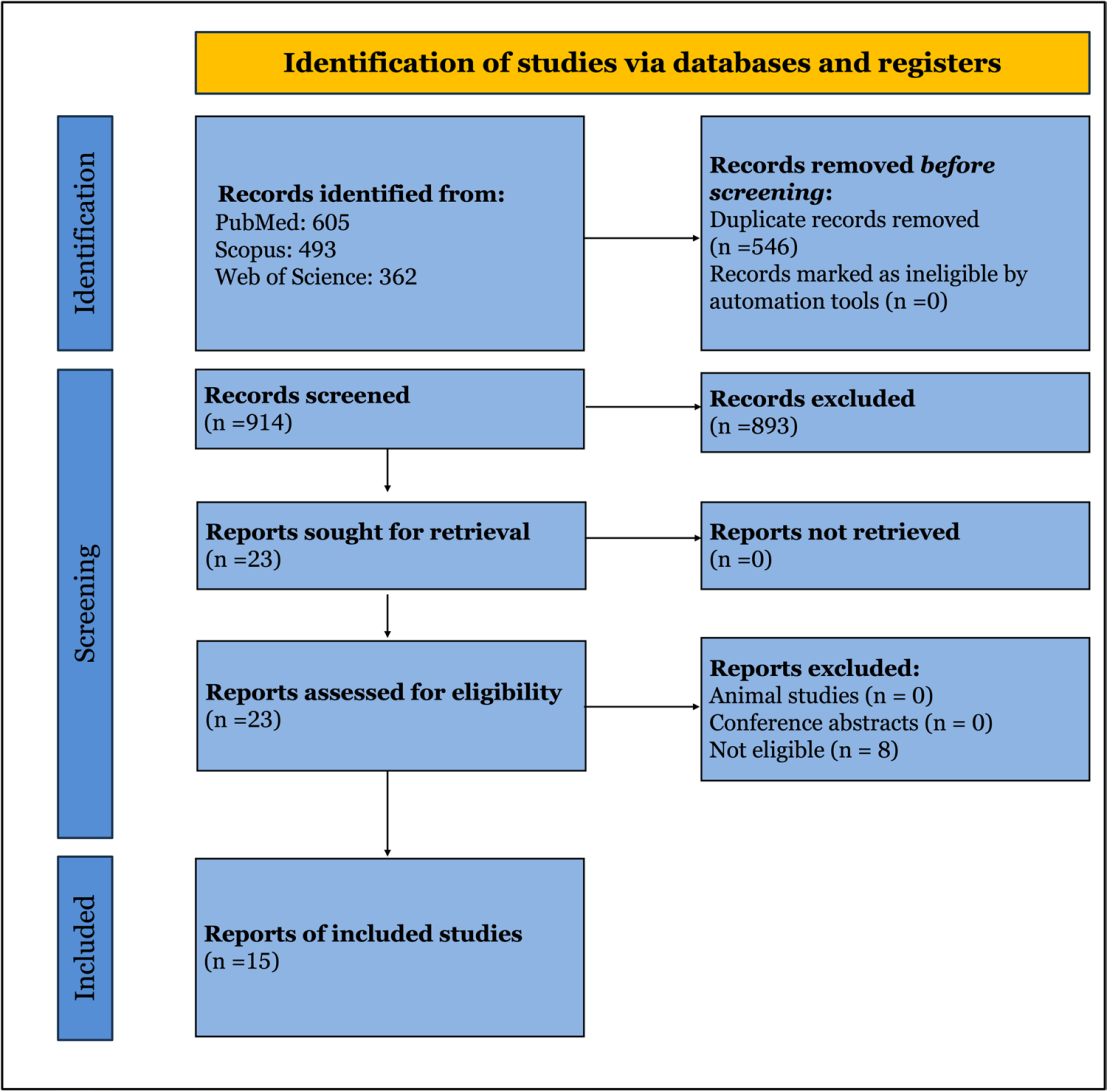
PRISMA flow chart

Our search yielded a total of 1460 articles, 605 from PubMed, 362 from WOS, and 493 from Scopus. After removing 546 duplicates, title and abstract screening was performed on 914 articles to exclude 893 more. After full-text screening, 15 RCTs were included in this meta-analysis PRISMA Fig. 1. These studies included a total of 2233 patients with a follow-up duration ranging from 1.5 to 5 years. 10 studies [7, 25–37] included patients who had GIT surgeries, and five studies [15, 26, 38–40] included patients having open AAA surgery. A total of 13 studies (low and some concerns ROB) were included in the final analysis, and the remaining 2 studies [27, 28, 32] were identified as high risk of bias, so they were excluded from the analysis to yield high-quality evidence-based results. The characteristics of the included studies are found in Table 1.

The mean age of the participants ranged from 36.8 ± 0.9 to 74.3 ± 5.8 years. The mean BMI ranged from 25.8 ± 4.96 to 52.23 kg/m^2^. The mean surgery time ranged from 117.83 ± 72.2 to 293 ± 119 min. The participants’ demographics are presented in Table 2. Additionally**,** Table 3 shows a collection of GIT causes of laparotomy.

**Table 2:** Baseline characteristics of the included studies. *NA* not applicable*, SD* standard deviation

Study ID	Arm	Number of participants *n*(%)	Age of participants mea*n* (SD)	Sex *n*(%)		BMI mea*n* (SD)	Smoking *n*(%)	Previous laparotomy *n*(%)	History of previous hernia *n*(%)	Blood transfusion mean (SD)	Surgery time (min) mea*n* (SD)	Diabetes *n*(%)	Cardiovascular diseases *n*(%)	COPD *n*(%)
				Males	Females									
Abo-ryia 2013	Mesh	32 (50%)	38.5 ± 10.8	6(18.8%)	26(81.3%)	52.2 ± 9.1	NA	NA	NA	NA	NA	NA	NA	NA
	No mesh	32 (50%)	36.9 ± 11.3	7(28%)	25(78.13%)	51.4 ± 10.5	NA	NA	NA	NA	NA	NA	NA	NA
Bali 2014	Mesh	20 (50%)	74.3 ± 5.8	18 (90%)	2(10%)	25.4	NA	NA	NA	NA	181 ± 38	4 (20%)	NA	10 (50%)
	No mesh	20 (50%)		18 (90%)	2(10%)	24.4	NA	NA	NA	NA	131 ± 27	6 (30%)	NA	7 (35%)
Bevis 2010	Mesh	40 (47.1%)	74 ± 6.25	34(85%)	6(15%)	NA	NA	NA	8 (20%)	NA	153.75 ± 33.75	6 (15%)	26 (65%)	NA
	No mesh	45 (52.9%)	72 ± 7.5	43(95.6%)	2(1.04%)	NA	NA	NA	13 (28.9%)	NA	167.5 ± 52.5	4 (8.9%)	28 (62.2%)	NA
Brosi 2017 & Glauser 2019	Mesh	131 (49%)	64.1 ± 13.14	60(45.8%)	71(54.2%)	25.8 ± 4.96	43 (32.8%)	16 (12.2%)	NA	NA	282 ± 105.11	13 (9.9%)	*10 (7.6%)*	NA
	No mesh	136 (51%)	65.1 ± 13.03	56(41.2%)	80(58.8%)	26.6 ± 4.76	42 (30.9%)	21 (15.4%)	NA	NA	*293* ± *119*	14 (10.3%)	*11 (8.1%)*	NA
Caro 2014 & Caro 2018	Mesh	80 (50%)	64.32 ± 14.27	44 (55%)	36 (45%)	NA	NA	NA	NA	NA	133.58 ± 50.4	13 (16.3%)	18 (22.5%)	19 (23.8%)
	No mesh	80 (50%)	67.32 ± 11.11	46 (57.5%)	34 (42.5%)	NA	NA	NA	NA	NA	117.83 ± 72.2	14 (17.5%)	24 (30%)	16 (20%)
El-khadrawy 2009	Mesh	20 (50%)	47.86 ± 13.82	10 (50%)	10 (50%)	NA	NA	NA	NA	NA	NA	4 (20%)	3 (15%)	NA
	No mesh	20 (50%)	47.61 ± 14.11	8(40%)	12 (60%)	NA	NA	NA	NA	NA	NA	4 (20%)	5 (25%)	NA
Garcia-urena 2015	Mesh	53 (49.8%)	65.6 ± 13.3	31 (58.5%)	22 (41.5%)	NA	5 (9.4)	8 (15.1%)	NA	17 (32.1%)	174.6 ± 65.8	18 (34%)	NA	NA
	No mesh	54 (50.2%)	61.46 ± 15.6	33 (61.1%)	21 (38.9%)	NA	9 (16.7)	13 (24.1%)	NA	10 (18.5%)	157.43 ± 82.8	9 (16.7%)	NA	NA
Gutierrez 2003	Mesh	50 (50%)	average age was 64.3 (range of 42–83)	67(67%)	33 (33%)	NA	NA	NA	NA	NA	NA	NA	NA	NA
	No mesh	50 (50%)				NA	NA	NA	NA	NA	NA	NA	NA	NA
Honig 2021	Mesh	34 (32.6)	70.53 ± 7.80	33 (97.1%)	1 (2.9%)	26.58 ± 4.04	20 (58.8%)	NA	NA	NA	NA	0 (0.0%)	17 (51.5%)	4 (12.5%)
	No mesh	35 (33.7%)	67.37 ± 9.55	32 (91.4%)	3 (8.6%)	26.82 ± 2.94	13 (38.2%)	NA	NA	NA	NA	2 (5.9%)	27 (77.1%)	7 (20%)
Jairam 2017	Onlay Mesh	188 (39.3%)	64·2 ± 12·3	116 (62%)	72 (38%)	30·8 ± 5.9	41 (22%)	10 (5%)	19 (10%)	NA	NA	36 (19%)	NA	24 (13%)
	No mesh	107 (22.2%	65·2 ± 10·5	68 (64%)	39 (36%)	29·8 ± 4·4	17 (16%)	3 (3%)	13 (12%)	NA	NA	19 (18%)	NA	9 (8%)
Kohler 2019	Mesh	69 (46%)	*66* ± *10.6*	46 (66.7%)	23(33.3%)	27.6 ± 4.6	NA	51 (73.9%)	NA	NA	275 ± 102	NA	NA	NA
	No mesh	81 (54%)	*64.1* ± *10.2*	56 (69.1%)	25 (30.9%)	26.7 ± 4.8	NA	60 (74.1%)	NA	NA	293 ± 109	NA	NA	NA
Muysoms 2016	Mesh	56 (49.1%)	72 ± 7.4	54 (96%)	2 (4%)	25 ± 3.6	35 (66%)	2 (4%)	*14 (25%)*	NA	211 ± 62	9 (17%)	NA	15 (27%)
	No mesh	58 (50.9%)	72 ± 8.5	52 (88%)	7 (12%)	26 ± 3.7	34 (63%)	0	10 (18%)	NA	190 ± 83	10 (18%)	NA	19 (35%)
Pans 1998	Mesh	144 (50%)	36.6 ± 0.9	41 (28.5%)	103 (71.5%)	43.8 ± 0.5	NA	NA	NA	NA	NA	18 (12.5%)	NA	NA
	No mesh	144 (50%)	36.4 ± 0.9	30 (20.8%)	114 (79.2%)	43.7 ± 0.6	NA	NA	NA	NA	NA	18 (12.5%)	NA	NA
Sarr 2014	Mesh	185 (48.7%)	44.6 ± 10.6	29 (21%)	110 (79%)	48.2 ± 8.2	NA	NA	NA	NA	NA	NA	NA	NA
	No mesh	195 (50.3%)	45.1 ± 12.1	28 (20%)	113 (80%)	48.2 ± 7.7	NA	NA	NA	NA	NA	NA	NA	NA
Strzelczyk 2006	Mesh	36 (48.6%)	39·4 ± 12·3	24 (66.7%)	12 (33.3%)	46·2 ± 7·1	NA	NA	NA	NA	NA	NA	NA	NA
	No mesh	38 (51.4%)	38·9 ± 11·8	23 (60.5%)	15 (39.5%)	46·8 ± 7·6	NA	NA	NA	NA	NA	NA	NA	NA

**Table 3:** GIT causes of laparotomy. *SD* standard deviation

Study	Arms	Anatomical Site					Etiology	
		Upper GI (Esophagus-1st part of duodenum) surgeries	Lower GI (2nd half of duodenum- anus) surgeries	Hepatobiliary surgeries	Pancreatic surgeries	Others (including bariatric surgeries)	Neoplasm	No neoplasm
Abo-ryia 2013	Mesh	0	0	0	0	32(50%)	0	32(50%)
	no mesh	0	0	0	0	32(50%)	0	32(50%)
Brosi 2017 & Glauser 2019	Mesh	NA	NA	NA	NA	NA	NA	NA
	no mesh	NA	NA	NA	NA	NA	NA	NA
Caro 2014 & Caro 2018	Mesh	33(41.25%)	40(50%)	0	0	7(8.75%)	58(72.5%)	22(27.5%)
	no mesh	15(18.75%)	63(78.755)	0	0	2(2.5%)	72(90)	8(10)
El-khadrawy 2009	Mesh	NA	NA	NA	NA	NA	NA	NA
	no mesh	NA	NA	NA	NA	NA	NA	NA
Garcia-urena 2015	Mesh	0	53 (49.8%)	0	0	0	45(84.9%)	8(16.1%)
	no mesh	0	54 (50.2%)	0	0	0	39(72.7%)	15(27.3%)
Gutierrez 2003	Mesh	7(14%)	39(78%)	3(6%)	1(2)	0	37(74%)	13(26%)
	no mesh	5(10%)	39(78%)	5(10%)	1(2)	0	39(78%)	11(22%)
Kohler 2019	Mesh	14(20.3%)	17(24.9%)	20(29%)	15(21.7%)	3(4.3%)	54(78.3%)	15(21.2%)
	no mesh	12(14.8%)	19(23.5%)	18(22.2%)	30(37%)	2(2.5%)	67(82.7%)	
Pans 1998	Mesh	0	0	0	0	144 (100%)	0	144 (100%)
	no mesh	0	0	0	0	144 (100%)	0	144 (100%)
Sarr 2014	Mesh	0	0	0	0	185 (100%)	0	185 (100%)
	no mesh	0	0	0	0	195 (100%)	0	195 (100%)
Strzelczyk 2006	Mesh	0	0	0	0	36 (100%)	0	36 (100%)
	no mesh	0	0	0	0	38 (100%)	0	38 (100%)

### Risk of bias and grade assessment

**Fig. 2:**
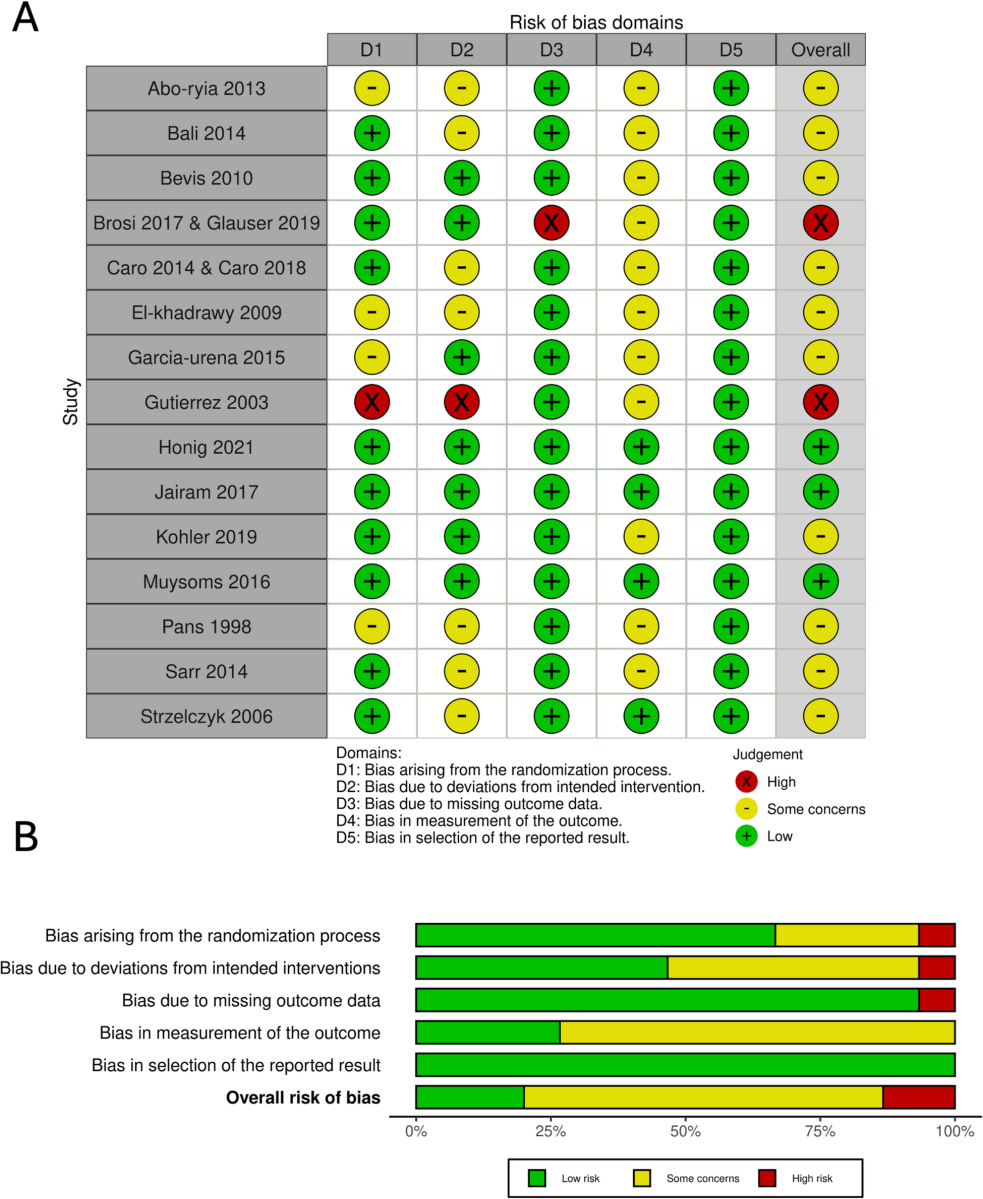
**A** The bias-risk assessment diagram and **B** summary of the included articles

The 15 included RCTs were assessed for the risk of bias using the ROB 2 tool. As a result, 10 studies were classified as having some concerns. Three studies showed low risk of bias, while two studies showed high risk of bias [27, 28, 32], mainly due to bias arising from the randomization process and missing outcome data as shown in Fig. 2.

A GRADE assessment was performed to evaluate the certainty of evidence for key outcomes. For open AAA repairs, prophylactic mesh reinforcement demonstrated a high certainty of evidence in reducing IH incidence across all follow-up periods (12, 24, and 36 months). These findings are detailed in ESM 3.

For GIT elective surgeries, mesh reinforcement showed a moderate certainty of evidence at 12 and 24 months due to heterogeneity, while the certainty decreased to low and very low at 36 and 48 months, respectively, primarily due to imprecision and inconsistency, as shown in ESM 4.

### Primary outcomes

#### IH after open AAA

**Fig. 3:**
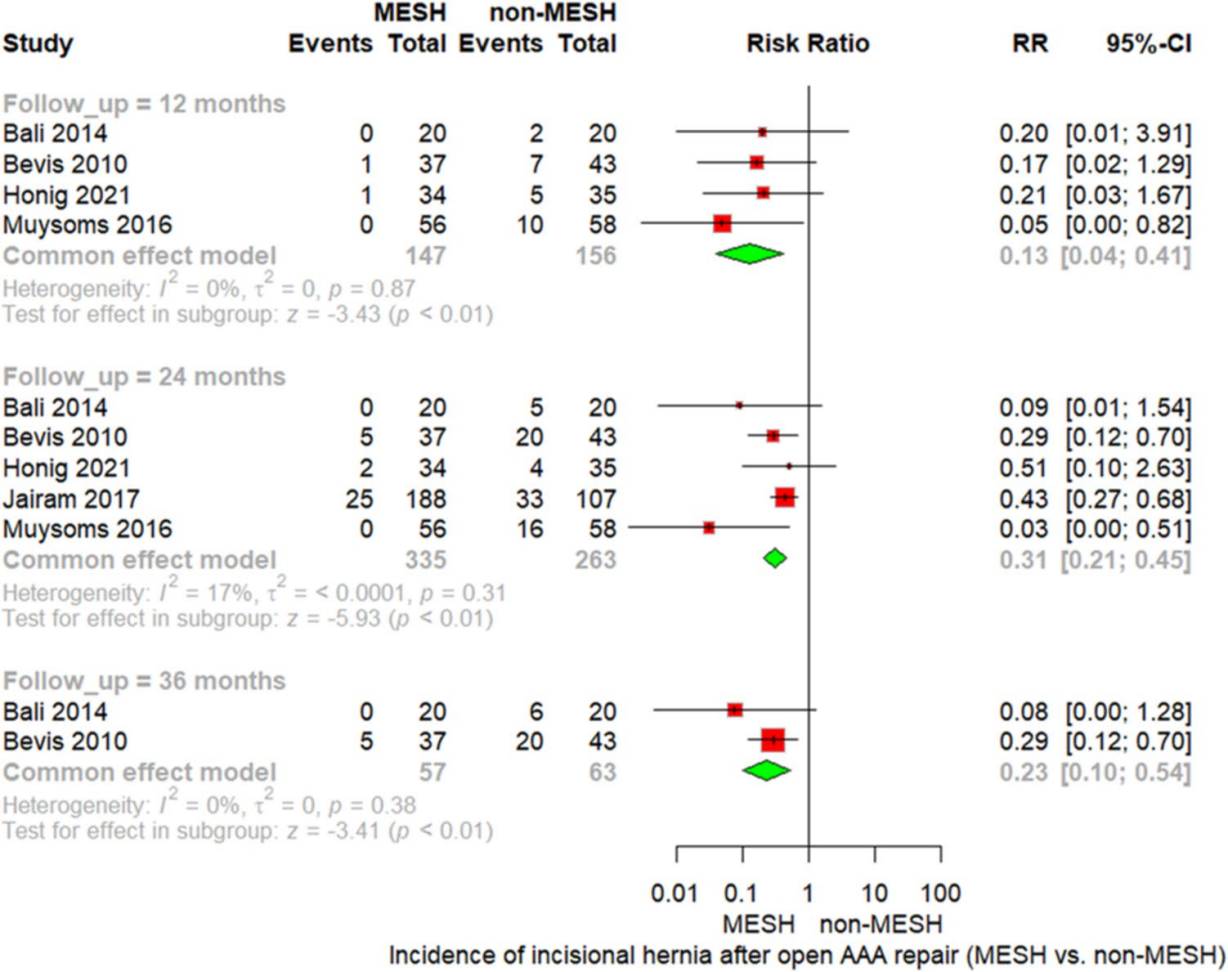
Forest plot of IH after open AAA

Our meta-analysis demonstrated that prophylactic mesh reinforcement significantly reduced the incidence of IH following open AAA repair across all reported follow-up periods. At 12 months, the pooled RR was 0.13 (95% CI [0.04; 0.41], *p* < 0.01). At 24 months, the effect remained significant with a pooled RR of 0.31 (95% CI [0.21; 0.45], *p* < 0.01). At 36 months, mesh reinforcement continued to be associated with a significantly lower risk of IH (RR = 0.23, 95% CI [0.10; 0.54], *p* < 0.01), with no observed heterogeneity at all time points. As shown in Fig. 3.

#### IH after GIT causes

**Fig. 4:**
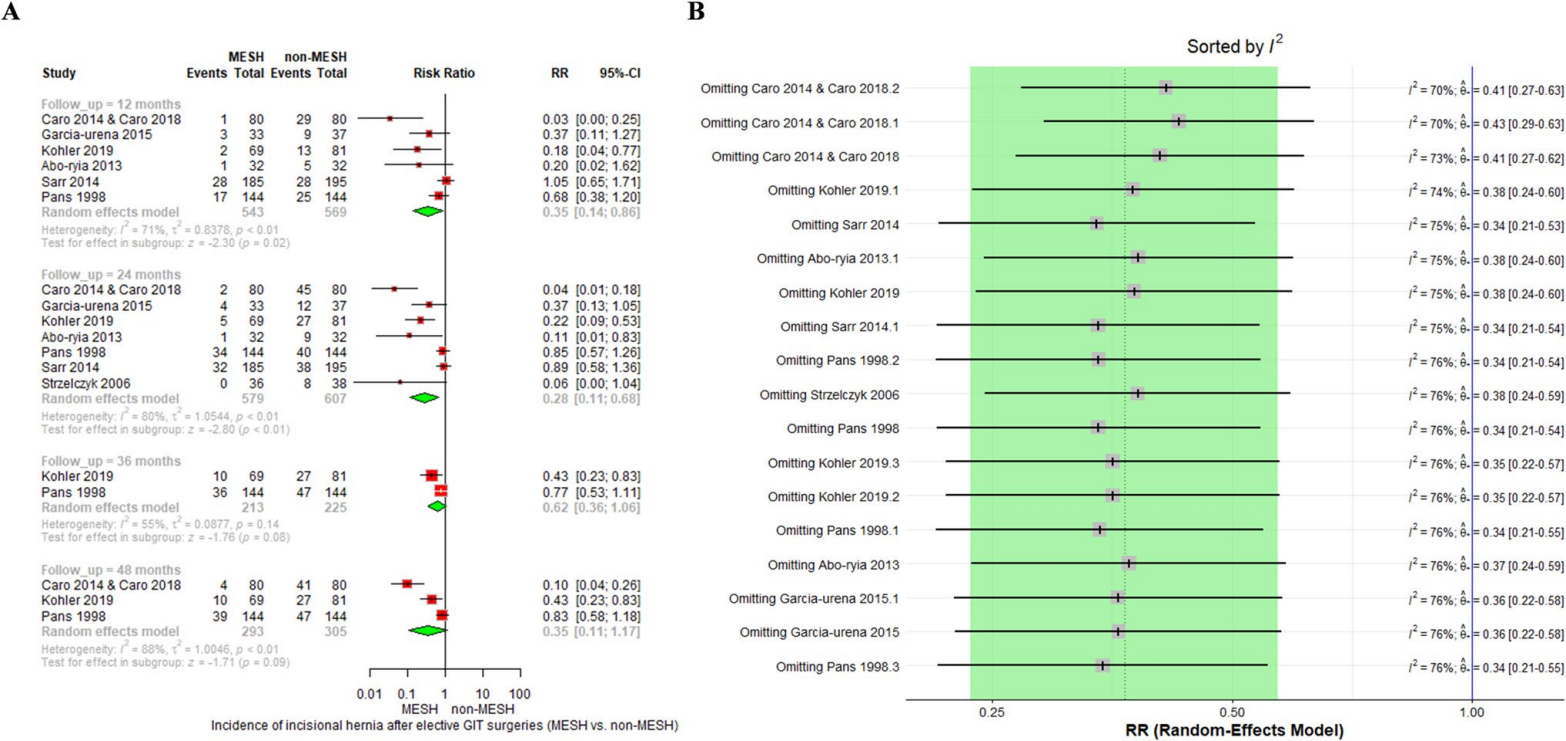
**A** shows the overall forest plot for IH after elective GIT surgeries, **B** shows the leave-one-out sensitivity analysis

Our meta-analysis demonstrated that prophylactic mesh reinforcement significantly reduced the incidence of IH following elective GIT surgeries. At 12 months, the pooled RR was 0.35 (95% CI [0.14; 0.86], *p* = 0.02); at 24 months, RR = 0.28 (95% CI [0.11; 0.68], *p* < 0.01); at 36 months, RR = 0.62 (95% CI [0.36; 1.06], *p* = 0.08); and at 48 months, RR = 0.35 (95% CI [0.11; 1.17], *p* = 0.09), as shown in Fig. 4A.

Substantial heterogeneity was observed across timepoints (I2 = 71–88%). Attempts to address this by switching from fixed-effect to random-effects models and conducting sensitivity analyses—including exclusion of low-BMI, high-BMI, and specific studies, did not resolve heterogeneity, as shown in Fig. 4B. Despite this, the protective effect of mesh remained directionally consistent across all analyses.

### Secondary outcomes

Mesh reinforcement significantly reduced re-operation after IH in elective GIT surgeries. Onlay placement showed a significant reduction (RR = 0.33, 95% CI [0.14; 0.79], *p* = 0.01) with moderate heterogeneity (I^2^ = 71%, *p* = 0.06), while Sublay also showed benefit (RR = 0.30, 95% CI [0.11; 0.81], *p* = 0.02) with low heterogeneity (I^2^ = 14%, *p* = 0.31).

**Fig. 5:**
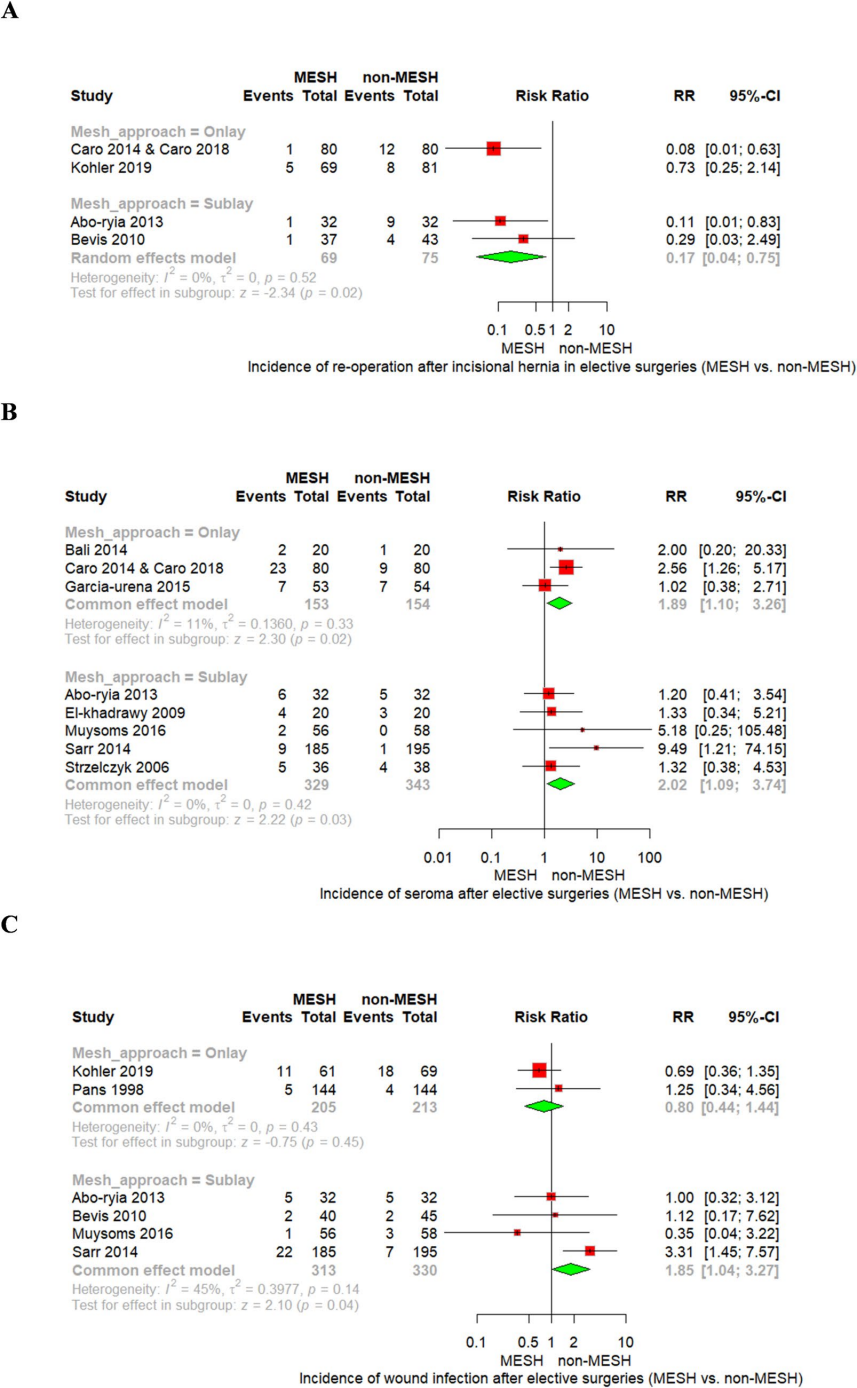
Forest plot of **A** re-operation after IH, **B** seroma, and **C** wound infection

To address heterogeneity, a random model was applied in a sensitivity analysis. Excluding Kohler 2019 (higher BMI, broader criteria) yielded stronger effect estimates in Onlay (RR = 0.08, 95% CI [0.01; 0.63]) and resolved heterogeneity in Sublay (I^2^ = 0%, *p* = 0.52). The findings are shown in Fig. 5A, supporting the consistent benefit of mesh across all approaches.

Complication profiles differed by mesh technique. Sublay placement was associated with significantly higher risks of both seroma (RR = 2.02, 95% CI [1.09; 3.74], *p* = 0.03) and wound infection (RR = 1.85, 95% CI [1.04; 3.27], *p* = 0.04). Onlay mesh was also linked to increased seroma (RR = 1.89, 95% CI [1.10; 3.26], *p* = 0.02), but not wound infection (RR = 0.80, 95% CI [0.44; 1.44], *p* = 0.45). No significant heterogeneity was observed across these outcomes, as shown in Fig. 5B and C, respectively.

**Fig. 6:**
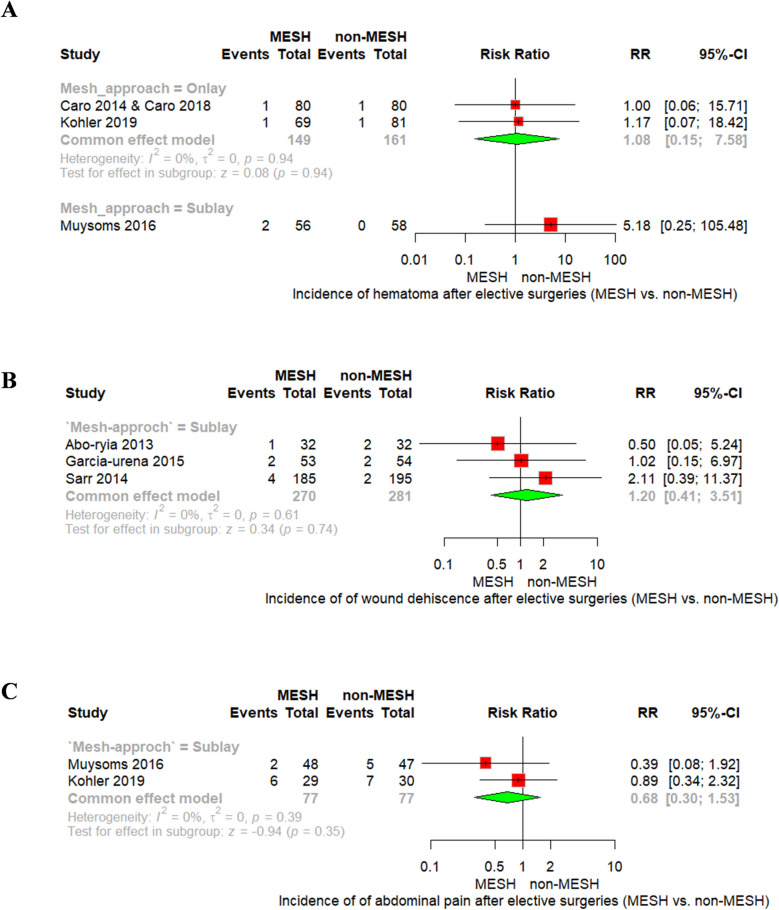
Forest plot of **A** hematoma, **B** wound dehiscence, and **C** abdominal pain

**Fig. 7:**
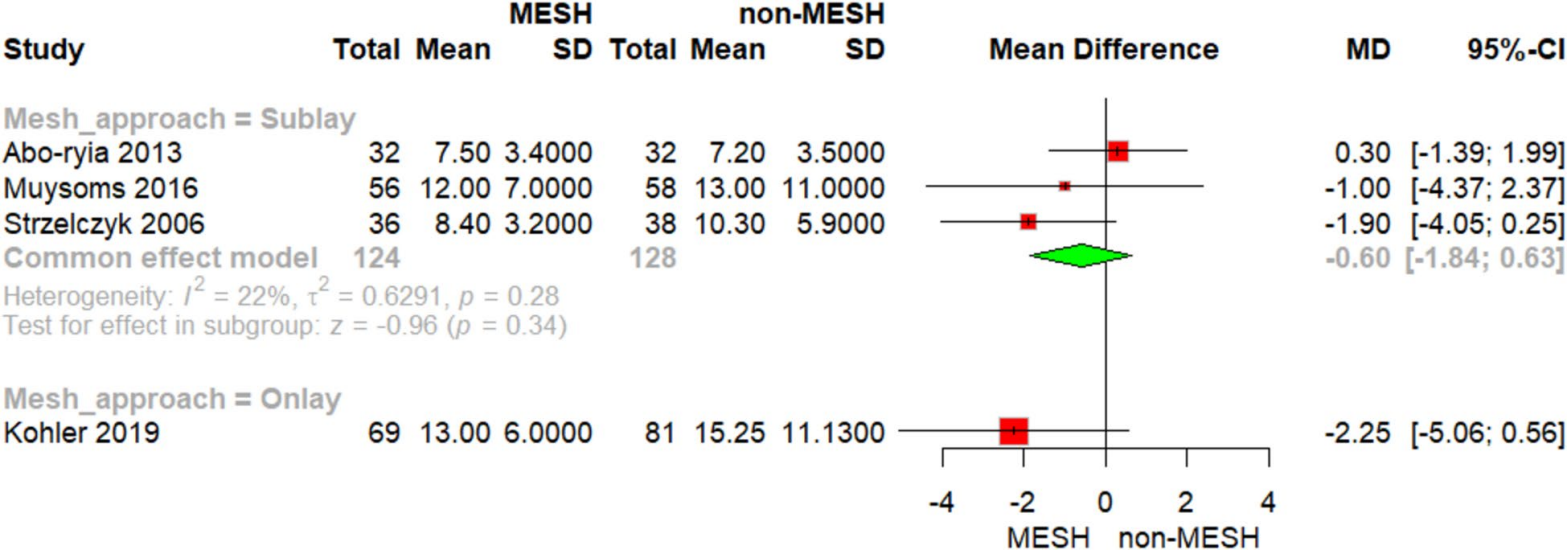
Forest plot for duration of hospitalization

No significant differences were observed in the incidence of hematoma with either onlay mesh placement (RR = 1.08, 95% CI [0.15; 7.58], *p* = 0.94) or sublay mesh placement (RR = 5.18, 95% CI [0.25; 105.48]), Fig. 6A. Similarly, wound dehiscence, reported exclusively in sublay mesh studies, showed no significant difference between groups (RR = 1.20, 95% CI [0.41; 3.51], *p* = 0.74), Fig. 6B. For abdominal pain, also examined in sublay studies, the difference was not significant (RR = 0.68, 95% CI [0.30; 1.53], *p* = 0.35), Fig. 6C. All outcomes demonstrated no evidence of heterogeneity. Also, Sublay mesh resulted in insignificant results regarding duration of hospitalization (MD = −0.60, 95% CI [−1.84 to 0.63], P = 0.34), with non-significant heterogeneity as shown in Fig. 7.

## Discussion

Laparotomy performed as elective abdominal surgery accounts for a large fraction of laparotomy procedures performed worldwide [41]. Although surgical techniques continue to advance, the incidence of IH remains one of the most common and serious postoperative complications, and occurs in as many as 20% of patients [42]. Millions of elective surgeries occur throughout the United States and globally every year, and at least one-fifth of these procedures will encounter some sort of issue with wound healing, and many will eventually lead to an IH [41–43], which may further become symptomatic, and in worst case scenarios, cause emergency complications including incarceration or strangulation, as a consequence of delayed care in low-and middle-income countries is concerning, due to lack of access to surgical care, poor infrastructure, and delays to emergency medicine care that may worsen the outcomes and morbidity and mortality from untreated hernias [44]. Improving long-term surgical outcomes with fewer adverse events (morbidity) for patients through minimizing or preventing IH is important, intending to reduce the burdens placed on populations requiring surgical intervention and reducing the technical intricacies and risks of repairing an IH if the need arises.

In this meta-analysis of 15 RCTs that enrolled 2233 patients, there was a statistically significant association between prophylactic mesh reinforcement during elective abdominal surgery with decreased rates of IH in both gastrointestinal and vascular procedures at all follow-ups. Prophylactic mesh reinforcement significantly decreased reoperation rates for hernias, with Onlay and Sublay procedures equally beneficial. However, mesh reinforcement increased seroma formation risk and the risk of wound infection when the mesh was placed in the Sublay position. There was no significant difference in the rate of hematoma formation, dehiscence, abdominal pain, or length of stay between mesh and non-mesh groups. Overall, complication onset was slightly increased using mesh; however, mesh reinforcement markedly improved hernia-related outcomes.

Despite the significant reduction in IH rates observed with prophylactic mesh reinforcement in elective GIT surgeries, substantial heterogeneity was present across studies. We conducted a series of sensitivity analyses, including exclusion of studies involving distinct populations (e.g., bariatric patients, those with high seroma rates), stratification by patient BMI, and leave-one-out analyses, but heterogeneity persisted. While the direction and statistical significance of the pooled effect remained consistent, the magnitude of heterogeneity suggests underlying variability in clinical practice. These differences likely reflect variation in mesh type, surgical technique, and patient characteristics such as BMI. Therefore, although mesh reinforcement appears consistently protective, these findings should be interpreted cautiously, with greater emphasis placed on the direction of effect rather than the precise pooled estimates.

Our findings are in broad agreement with previous meta-analyses by Frassini et al. [45] and Valério-Alves et al. [46], confirming that prophylactic mesh reinforcement significantly reduces the incidence of IH following elective abdominal surgeries. Both of the previous analyses reported similar outcomes, concluding that mesh placement could be used to improve surgical outcomes and have no major complications (wound infection, hematoma, or hospital stay) of increased importance. There were, however, some subtle differences. Each of the three analyses reported a higher wound infection with Sublay mesh specifically; our study offered more detail in this area, as Frassini et al. [45] do not highlight any statistically significant risk of infection, while Valério-Alves et al. [46] reported no clinically relevant difference by technique. On the seroma risk, while all studies indicated an increased risk of seroma, we reported a stronger association with Sublay placement. Our reported apparent differences in risk may be explained by differences in surgical technique, risk profiles among patients, or length of follow-up in the included trials. Our cause-specific analysis by surgical indication, GIT vs. open AAA, is also an important advantage of our analysis as it facilitates further granularity in identifying from the clinical context if any prophylactic mesh reinforcement as a form of IH is effective or safer, and this important aspect was not addressed in previous reviews. Our results also align closely with the updated meta-analysis by Hew et al. [47], which focused specifically on elective open AAA repairs. Hew et al. reported a significant reduction in IH rates with prophylactic mesh (OR 0.20, 95% CI 0.09–0.43), consistent with our findings in vascular surgery patients. Furthermore, Hew et al. found no significant increase in wound infections, matching the infection risk profile observed in our analysis and those of Frassini and Valério-Alves. However, unlike Hew’s study, which focused only on vascular surgery and applied trial sequential analysis to confirm that no further RCTs are needed, our analysis included a broader elective surgery population (both GIT and open AAA cases) and examined mesh positioning effects. The agreement across studies reinforces the growing evidence base supporting mesh reinforcement in elective laparotomies, while the observed variations emphasize the need for individualized surgical strategies. A summary of our study’s results and Hew et al., Frassini et al., and Valério-Alves et al. results is shown in Table 4. Although we acknowledge that mesh material type (e.g., synthetic vs. biologic; permanent vs. absorbable) may impact surgical outcomes, a direct comparative analysis was not conducted due to limited and heterogeneous data. However, this remains a relevant consideration and merits further investigation in future studies. ESM. 2 provides a detailed summary of the mesh types used in each included study. Moreover, in both the Bali et al. [39] and Muysoms et al. [38] studies, which included patients undergoing open AAA repair, operative time was significantly longer in the mesh groups compared to the suture-only groups. Bali et al. reported a mean operative time of 181 ± 38 min in the mesh group versus 131 ± 27 min in the control group (*p* < 0.001), while Muysoms et al. observed 164 ± 58 min versus 139 ± 50 min (*p* < 0.0001). Despite the increased duration, neither study found a corresponding rise in postoperative morbidity, supporting the clinical acceptability of the additional time required for prophylactic mesh placement.

**Table 4:** Summary of other studies’ data in comparison to our data. *SD* standard deviation, *AAA* abdominal aortic aneurysm, *IH* incisional hernia

	Frassini et al	Hew et al	Valério-Alves et al	Our analysis
Number of studies included	18 studies	5 studies	15 studies	15 studies
Number of patients	2553 patients	487 patients	2108 patients	2233 patients
Type of laparotomies	Elective and emergent laparotomy	Elective open AAA	Elective and emergent laparotomy	Elective laparotomy
IH outcome	-Mesh reduced IH incidence at 1, 2, 3, and 4 years -No specification of the cause of laparotomy	-Mesh reduced IH incidence -No specification of the years of follow up	-Mesh reduced IH incidence -No specification of the years of follow up -No specification of the cause of laparotomy	-IH was reduced after open AAA at 1, 2, and 3 years -IH was reduced after GIT at 1, 2, 3, and 4 years
Seroma	Was higher in non-mesh group	NA	Was higher in non-mesh group	-Sublay and Onlay mesh placement increased seroma incidence
Hematoma	NA	NA	Results were not significant	Results were not significant
Re-operation for IH	NA	Was higher in non-mesh group	NA	Sublay and Onlay mesh shwed reduction in re-operation incidence
Wound infections	Results were not significant	Results were not significant	Results were not significant	-Sublay mesh placement increased wound infections
Duration of hospitalization	NA	NA	Results were not significant	Results were not significant
Wound dehiscence	Results were not significant	NA	Results were not significant	Results were not significant
Abdominal pain	NA	NA	Results were not significant	Results were not significant

### Strengths, limitations, and implications for future research

This meta-analysis provides good evidence in favor of the use of prophylactic mesh reinforcement in elective abdominal surgery. It included a large sample of RCTs with long follow-up durations, subgroup analysis that facilitated detailed cause-specific investigations, and an analysis of mesh placement strategies. Nevertheless, the meta-analysis is subject to limitations, including moderate-to-high heterogeneity in gastrointestinal surgery outcomes, differences in mesh composition and methods of fixation, and the inclusion of some studies at risk of bias. Nevertheless, the applicability of the findings should be considered limited to elective surgeries, and the evidence may not have direct applicability to emergency surgery. Additionally, the exclusion of non-English studies may have introduced language bias. Future research should work to facilitate standardized comparisons of mesh types and mesh placement, follow patients for longer than five years post-operatively, develop risk stratification modeling for optimization of mesh use, conduct studies evaluating cost-effectiveness, and consider future studies on patient-centered outcomes to provide informed surgical practice.

## Conclusion

Prophylactic mesh reinforcement during elective abdominal surgery significantly reduces the risk of IH and the need for reoperation. However, it is associated with an increased risk of seroma formation and, to a lesser extent, wound infection, particularly with the Sublay technique. Mesh placement does not significantly affect the incidence of hematoma, wound dehiscence, postoperative abdominal pain, or the length of hospital stay.

## Supplementary Information

Below is the link to the electronic supplementary material.

Supplementary file1 (PDF 308 KB)

## Data Availability

The datasets used and/or analyzed during the current study are available from the corresponding author on reasonable request. All data relevant to the study are included in the article and its supplementary materials. Additional data can be provided upon reasonable request to the corresponding author.

## Declarations

All authors have shared in the final drafting of the manuscript.

All authors have read and approved the final manuscript.

### Submission declaration

We confirm that this manuscript has not been published elsewhere and is not under consideration by another journal. All authors have approved the manuscript and agree with its submission to *Hernia*.
