# Supplementary material for: Prophylactic mesh reinforcement in elective abdominal surgeries: a systematic review, meta-analysis, and GRADE evidence assessment
Source: Hernia. 2025 Jul 11;29(1):230. doi: 10.1007/s10029-025-03421-9 (PMC12254175; doi:10.1007/s10029-025-03421-9)
Supplement: Supplementary file 1 — Supplementary file1 (PDF 308 KB) [file 10029_2025_3421_MOESM1_ESM.pdf]

Electronic Supplementary Materials

Prophylactic Mesh Reinforcement in Elective Abdominal Surgeries: A Systematic Review, Meta-Analysis, and GRADE Evidence Assessment

Contents

ESM. 1 Specific Search Strategy for each database 2

ESM. 2 Mesh type and number of included studies 2

ESM. 3 GRADE Evidence Profile: Prophylactic Mesh Reinforcement vs Non-Mesh in Preventing Incisional Hernia After Open AAA Repair 3

ESM. 4 GRADE Evidence Profile: Efficacy of Prophylactic Mesh Reinforcement in Elective GIT Surgeries for Incisional Hernia Prevention 3

ESM. 1 Specific Search Strategy for each database

Database	Specific search strategy	Number of retrieved references
**PubMed**	(Mesh OR (Abdominal Wound Closure) OR net OR network) AND (Elective) AND ((laparotomy) OR Celiotomy OR (midline laparotomy) OR colorectal OR (abdominal cavity) OR (abdominal midline incision))	605
**Web of Science**		493
**Scopus**	("Mesh" OR ("Abdominal Wound Closure") OR "net" OR "network") AND ("Elective") AND (("laparotomy") OR "Celiotomy" OR ("midline laparotomy") OR "colorectal" OR ("abdominal cavity") OR ("abdominal midline incision"))	362

ESM. 2 Mesh type and number of included studies

Mesh Type	Number of Studies	Study References
**Polypropylene (non-absorbable synthetic)**	11	Abo-Ryia, Bevis, Caro, El-Khadrawy, García-Ureña, Gutiérrez, Honig, Jairam, Kohler, Muysoms, Strzelczyk
**Polyester (polyethylene terephthalate) (non-absorbable synthetic)**	1	Brosi & Glauser
**Polyglactin (absorbable synthetic)**	1	Pans
**Monofilament polyester + resorbable polylactic acid gripping system (composite)**	1	Pizza
**Bovine pericardium (biologic)**	1	Bali
**Surgisis Gold graft (biologic – porcine submucosa)**	1	Sarr

ESM. 3 GRADE Evidence Profile: Prophylactic Mesh Reinforcement vs Non-Mesh in Preventing Incisional Hernia After Open AAA Repair

Author(s):
Question: Prophylactic mesh reinforcement compared to Non-Mesh for educing incisional hernia in adults undergoing elective abdominal surgery (including gastrointestinal and open abdominal aortic aneurysm repair)
Setting:
Bibliography:

Certainty assessment							№ of patients		Effect		Certainty	Importance
№ of studies	Study design	Risk of bias	Inconsistency	Indirectness	Imprecision	Other considerations	Prophylactic mesh reinforcement	Non-Mesh	Relative (95% CI)	Absolute (95% CI)		
**Incidence of incisional hernia after open AAA repair - 12-month follow-up (follow-up: 12 months; assessed with: Incidence)**												
4	randomised trials	not serious	not serious	not serious	not serious[a]	none	147/303 (48.5%)	24/156 (15.4%)	**RR 0.13** (0.04 to 0.41)	**134 fewer per 1,000** (from 148 fewer to 91 fewer)	⊕⊕⊕⊕ High[a]	CRITICAL
**Incidence of incisional hernia after open AAA repair - 24-month follow-up (follow-up: 24 months; assessed with: Incidence)**												
5	randomised trials	not serious	not serious	not serious	not serious[b]	none	32/335 (9.6%)	72/263 (27.4%)	**RR 0.31** (0.21 to 0.45)	**189 fewer per 1,000** (from 216 fewer to 151 fewer)	⊕⊕⊕⊕ High[b]	CRITICAL
**Incidence of incisional hernia after open AAA repair - 36-month follow-up (follow-up: 36 months; assessed with: Incidence)**												
2	randomised trials	not serious	not serious	not serious	not serious[c]	none	5/57 (8.8%)	26/63 (41.3%)	**RR 0.23** (0.10 to 0.54)	**318 fewer per 1,000** (from 371 fewer to 190 fewer)	⊕⊕⊕⊕ High[c]	CRITICAL

CI: confidence interval; **RR:** risk ratio

Explanations

a. The 95% CI [0.04–0.41] is narrow, excludes no effect, and supports a clear benefit. The sample size is adequate to detect a meaningful difference.
b. The confidence interval (RR = 0.31, 95% CI [0.21 to 0.45]) is narrow and does not cross the line of no effect. The total sample size (N = 598) supports a precise, clinically meaningful benefit.
c. The 95% confidence interval (RR = 0.23, 95% CI [0.10 to 0.54]) is narrow, does not cross the line of no effect, and indicates a consistent benefit. Despite a smaller total sample size (N = 120), the effect is large and precise enough to support confident decision-making.

ESM. 4 GRADE Evidence Profile: Efficacy of Prophylactic Mesh Reinforcement in Elective GIT Surgeries for Incisional Hernia Prevention

Author(s):
Question: Prophylactic mesh reinforcement compared to Non-Mesh for educing incisional hernia in adults undergoing elective abdominal surgery (including gastrointestinal and open abdominal aortic aneurysm repair)
Setting:
Bibliography:

Certainty assessment							№ of patients		Effect		Certainty	Importance
№ of studies	Study design	Risk of bias	Inconsistency	Indirectness	Imprecision	Other considerations	Prophylactic mesh reinforcement	Non-Mesh	Relative (95% CI)	Absolute (95% CI)		
**Incidence of incisional hernia after elective GIT surgery - 12-month follow-up (follow-up: 12 months; assessed with: Incidence)**												
6	randomised trials	not serious	serious[a]	not serious[b]	not serious[c]	none	54/543 (9.9%)	106/569 (18.6%)	**RR 0.35** (0.14 to 0.86)	**121 fewer per 1,000** (from 160 fewer to 26 fewer)	⊕⊕⊕◯ Moderate[a,b,c]	CRITICAL
**Incidence of incisional hernia after elective GIT surgery - 24-month follow-up (follow-up: 24 months; assessed with: Incidence)**												
7	randomised trials	not serious	serious[d]	not serious	not serious[e]	none	46/394 (11.7%)	96/423 (22.7%)	**RR 0.28** (0.11 to 0.68)	**163 fewer per 1,000** (from 202 fewer to 73 fewer)	⊕⊕⊕◯ Moderate[d,e]	CRITICAL
**Incidence of incisional hernia after elective GIT surgery - 36-month follow-up (follow-up: 36 months; assessed with: Incidence)**												
2	randomised trials	not serious	serious[f]	not serious	serious[g]	none	46/213 (21.6%)	71/225 (31.6%)	**RR 0.62** (0.36 to 1.06)	**120 fewer per 1,000** (from 202 fewer to 19 more)	⊕⊕◯◯ Low[f,g]	CRITICAL
**Incidence of incisional hernia after elective GIT surgery - 48-month follow-up (follow-up: 48 months; assessed with: Incidence)**												
3	randomised trials	not serious	serious[h]	not serious	very serious[i]	none	53/293 (18.1%)	74/305 (24.3%)	**RR 0.35** (0.11 to 1.17)	**158 fewer per 1,000** (from 216 fewer to 41 more)	⊕◯◯◯ Very low[h,i]	CRITICAL

CI: confidence interval; **RR:** risk ratio

Explanations

a. High heterogeneity (I^2 = 71%)
b. Direct population and intervention
c. CI does not cross null and total N = 1112
d. High heterogeneity (I^2 = 80%)
e. Narrow CI, significant benefit, and sufficient total N
f. I^2 = 55% suggests moderate heterogeneity
g. CI crosses the line of no effect (includes both benefit and harm)
h. I^2 = 88% → high heterogeneity
i. CI is wide and crosses both sides of the line of no effect
